# Price Comparison of Human and Veterinary Formulations of Common Medications

**DOI:** 10.1001/jamainternmed.2022.3938

**Published:** 2022-09-12

**Authors:** Waqas Haque, Satheesh Chencheri, Beth A. Virnig, Anne H. Blaes, Christopher M. Booth, Stacie B. Dusetzina, Arjun Gupta

**Affiliations:** 1 Department of Internal Medicine, New York University Langone Health, New York, New York; 2 Banfield Pet Hospital, Minneapolis, Minnesota; 3 Division of Health Policy and Management, School of Public Health, University of Minnesota, Minneapolis; 4 Division of Hematology, Oncology and Transplantation, Department of Medicine, University of Minnesota Medical School, Minneapolis; 5 Department of Oncology, Queen’s University School of Medicine, Kingston, Ontario, Canada; 6 Cancer Care and Epidemiology, Cancer Research Institute, Queen’s University School of Medicine, Kingston, Ontario, Canada; 7 Department of Health Policy, Vanderbilt University Medical Center, Nashville, Tennessee; 8 Vanderbilt-Ingram Cancer Center, Nashville, Tennessee

## Abstract

This cross-sectional study compares prices of commonly prescribed medications used to treat both humans and pets.

In 2021, the US Food and Drug Administration oversaw the marketing of approximately 20 000 medications for human use and 1600 for veterinary use. Some medications are common to both pets and humans, and price differences can be extreme. In 1991, levamisole—introduced in the 1960s as a veterinary antiparasitic medication—demonstrated efficacy in treating human colon cancer. The introductory human price of Janssen’s Ergamisol (brand-name levamisole; $5 per 50-mg tablet) was 100 times the then veterinary price (approximately $0.05 for an equivalent amount).^1^ In 2021, demand for ivermectin for treatment of COVID-19, fueled by misinformation, led to people seeking veterinary formulations of the drug, increasing the price 15-fold over a month ($6 to $92 for 3 tubes).^2^ In this cross-sectional study, we sought to compare prices of commonly prescribed medications used to treat both humans and pets.

## Methods

We identified the 200 human medications with the most prescription fills using the ClinCalc database. For medications with the same ingredients also used in pets, we obtained the price per unit (eg, per tablet) in humans and pets. For human prices, we used GoodRx, a national-level price comparison website to calculate the average retail price (ARP) and a discounted price at Costco pharmacy for a typical fill of the most common human dosage.^3^ We obtained pet (dog) prices from online pharmacies via Google (eg, Chewy). We selected generic medications when available and human-equivalent doses (eg, lisinopril, 20 mg, in humans and pets). The primary outcome was the human-to-pet price ratio. Because this study involved secondary, deidentified data from a publicly available source, the University of Minnesota institutional review board considered this to be not human research and waived need for approval.

## Results

Of the 200 human medications identified, 120 (60.0%) with unique active ingredients and a pet formulation were studied. All medications except 1 (insulin detemir) had generic human formulations. The human ARP and discounted price was higher than the pet price for 112 (93.3%) and 77 (64.2%) medications, respectively.

**Figure 1.**
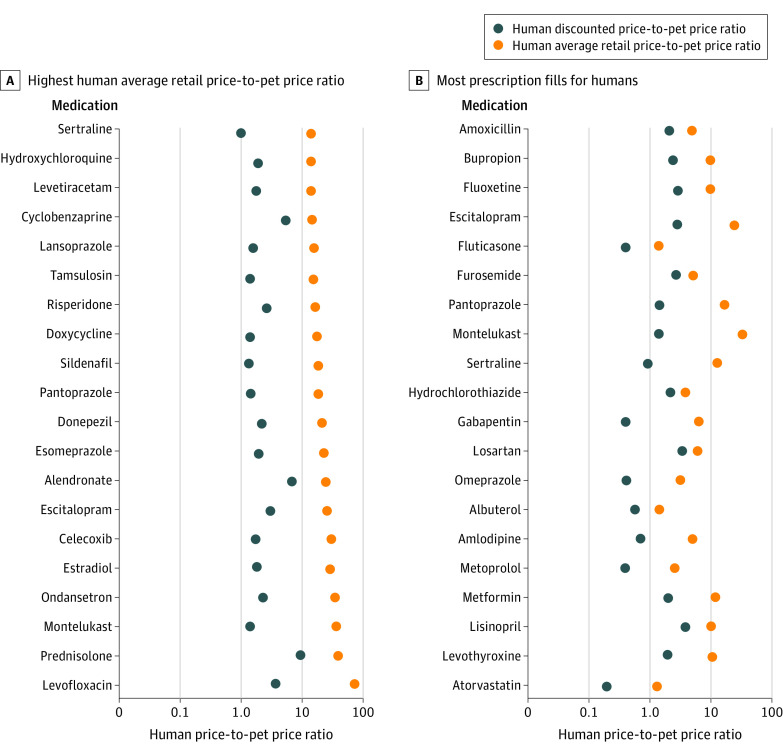
Human-to-Pet Per-Unit Price Ratios for Medications Human-to-pet per-unit price ratios for 20 medications with the highest human average retail price-to-pet price ratio (A) and 20 medications with the most prescription fills for humans (B). The x-axis is log_10_ scale.

**Figure 2.**
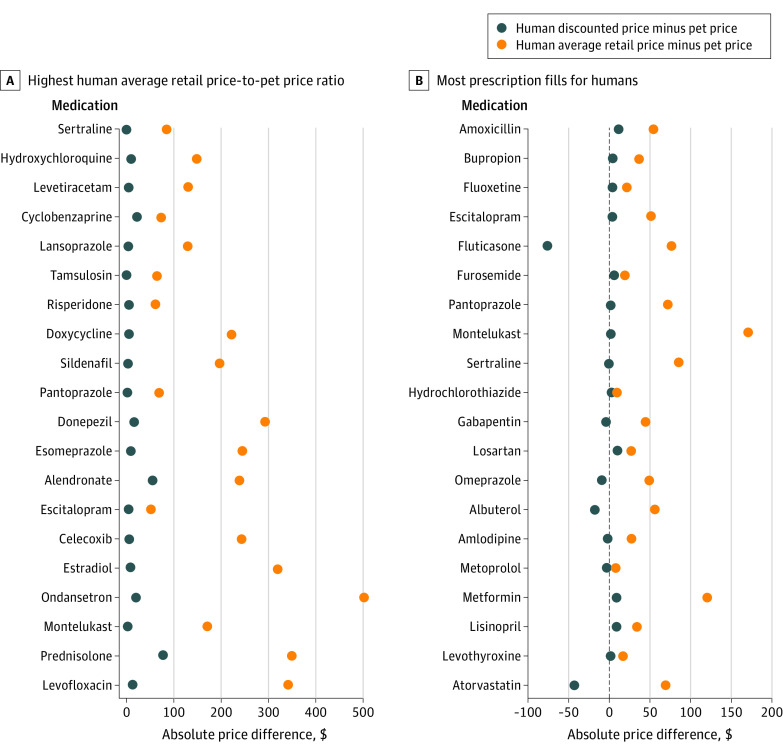
Absolute Price Differences Between Medications for Humans and for Pets Absolute price differences between prices for humans (average retail price and discounted price) and prices for pets for a 30-day fill among 20 medications with the highest human average retail price-to-pet price ratio (A) and 20 medications with the most prescription fills for humans (B).

The median (IQR) human ARP-to-pet price ratio was 5.5 (2.9-10.7), and the human discounted price-to-pet price ratio was 1.4 (0.7-2.5). The human ARP-to-pet price ratio was more than 10 for 35 (29.1%) medications. The human discounted price-to-pet price ratio was more than 3 for 20 (16.7%) medications (Figure 1). Figure 2 presents absolute differences in human and pet prices for a 30-day supply.

Of the medications studied, 15 (12.5%) were antimicrobials. The human ARP-to-pet price ratio was more than 1 for all antimicrobials, with a median of 4.4. The human discounted price-to-pet price ratio was more than 1 for 8 (53.3%) antimicrobials, with a median of 1.3.

## Discussion

In this cross-sectional study, we found that prices of most medications were higher for humans than for pets. Even discounted prices for humans, a best-case scenario of out-of-pocket costs for patients without prescription drug coverage, were higher than pet prices for two-thirds of medications.

Almost all medications were generics. Given that generic markets are more competitive than brand-name markets, price differences may reflect differences in manufacturing, regulatory standards, and distribution, as well as price discrimination (different prices in different markets with the same costs). Online pet pharmacies face less overhead in storage, and veterinary formulations may contain harmful (to humans) additives. Additionally, higher prices for humans may reflect pharmaceutical company investment, as well as differences in effectiveness and willingness to pay.^1^

Absolute price differences between human and pet prices for a 30-day supply were sometimes substantial, even for human discounted prices. A noteworthy example from 2018 involves a 5-mg tablet of phytonadione (oral vitamin K_1_) for humans costing $70.51, and a 50-mg veterinary-grade tablet costing $0.61.^4^

The human ARP of antimicrobials was 4 times the pet price. When antimicrobial access is appropriately limited through human sources by requiring a prescription, patients may turn to more accessible—and cheaper—pet antimicrobials.^5,6^

This work has limitations, including medication prices being dynamic, opaque rebates underlying discounted prices, and prices for humans often not being proportional to drug strength or fill quantity. Nonetheless, this study demonstrates that cash prices for generic medications should be transparent and accessible to people, for their own use and for their pets.
